# Effectiveness of emotion regulation strategies measured by self-report and EMG as a result of strategy used, negative emotion strength and participants’ baseline HRV

**DOI:** 10.1038/s41598-023-33032-2

**Published:** 2023-04-17

**Authors:** Dorota Kobylińska, Karol Lewczuk, Magdalena Wizła, Przemysław Marcowski, Christophe Blaison, Till Kastendieck, Ursula Hess

**Affiliations:** 1 Faculty of Psychology, University of Warsaw, Stawki 5/7, 00-183 Warsaw, Poland; 2 Institute of Psychology, Cardinal Stefan Wyszynski University, Warsaw, Poland; 3 Swartz Center for Computational Neuroscience, University of California, San Diego, San Diego, USA; 4 Institut de Psychologie, Université de Paris, Boulogne-Billancourt Cedex, France; 5 Department of Psychology, Humboldt-Universität of Berlin, Berlin, Germany

**Keywords:** Physiology, Psychology

## Abstract

We investigated how emotion regulation (ER) effectiveness—on both a self-reported rating as well as emotional expression (*corrugator supercilii* muscle activity) level—is affected by the characteristics of the situation (low *vs.* high negativity), the strategy used (reinterpretation, distraction, suppression, no regulation control condition) and individual dispositions (low *vs.* high baseline Heart Rate Variability) as well as their interaction. For this purpose, 54 adult women participated in a laboratory study. All the included factors significantly influenced both corrugator activity and appraisals of pictures’ negativity (in specific experimental conditions). For example, for high HRV participants, (1) distraction, suppression and reinterpretation significantly decreased corrugator activity compared to the control condition, and (2) distraction decreased appraised picture negativity for high negativity photos. For low HRV participants, distraction and suppression were most effective in decreasing corrugator responses, while suppression was more effective than reinterpretation in decreasing perceived picture negativity in the high negativity condition. Subjectively reported effort and success in applying ER strategies were also dependent on manipulated and dispositional factors. Overall, our results lend support to the flexible emotion regulation framework, showing that emotion regulation effectiveness relies on situational context as well as individual dispositions and their interaction.

## Introduction

Emotion regulation (ER) is critically important for psychological health, particularly when stress is high^1–9^. Emotion regulation (ER) deficits are associated with psychiatric symptoms^3,10^, whereas success in ER has many adaptive outcomes and correlates. These include better psychological health, increased well-being, better social functioning, more effective coping with stressful life events, and even better performance at school or in one’s job^11–13^. For this reason, research on the effectiveness of particular ER strategies has gathered significant attention from researchers, practitioners and the general public alike.

### Process model of emotion regulation

In his process model of ER, which seems the most seminal and dominating approach to ER, Gross^11,14^ defines ER as a set of processes by which individuals influence which emotions they have, when they have them, and how they experience and express them. These processes lead to changes to the dynamics, duration, and speed of emotion occurrence as well as changes in behavioral, experiential and physiological reactions elicited by an emotion. ER can be aimed at reducing, strengthening, or maintaining the experience of either positive or negative emotions depending on the current goals of an individual.

In the model, Gross describes five groups of ER strategies, related to the dynamics of emotional process: situation selection, situation modification, attention deployment (with distraction as one of the strategies), cognitive change (with reappraisal as one of the strategies) and response modulation (with expressive suppression as one of the strategies). The first four levels are classified as „antecedent-focused”, because they can be employed before the emotion response tendencies become fully activated^14^, thus enabling the change of an emotion itself. The last group of strategies is called „response-focused” strategies, as they are used after the emotion response tendencies have been generated and can influence the reactions initiated by the emotion. The three mentioned strategies, each being an example of a different group of ER strategies: distraction, reappraisal and expressive suppression, were chosen to be included in our study.

### Flexible emotion regulation

In recent years, it has been argued that the effectiveness of ER depends not only on the type of strategy applied but also on the interaction of the situation’s features and the individual characteristics of a person who regulates their emotions^2,15–18^. Although dynamic models have been proposed in which adaptive ER is based on dispositional as well as situational factors, most studies have relied on the assumption that specific ER strategies can be characterized as adaptive or maladaptive (irrespective of the context); this conviction has been referred to as the fallacy of uniform efficacy^19^.

In general, it is psychological flexibility that accounts for adaptive reactions^20,21^. In the domain of ER “[…] flexibility refers to the ability to implement ER strategies that are synchronized with contextual demands”^2^. In line with this proposition, better ER may result from using different strategies depending on contextual factors and individual characteristics, while psychological dysfunction may be characterized by deficits in flexibility^19,22,23^. Thus, research should focus on finding the best situation-strategy-fit patterns, showing which strategy may be most effective in a given situational context^9,15,19,24,25^. Research should also focus on the interaction of contextual and dispositional variables in predicting ER strategies' effectiveness.

### Importance of situational and dispositional characteristics

Even though the flexible ER framework holds a lot of promise, only a limited amount of research has focused on the interplay between contextual and individual factors^18^. Yet, the lack of consistency in the effectiveness of coping strategies in different situations and for different emotion intensity levels suggests that strategies are sensitive to contextual factors^26,27^. Indeed, some studies explicitly demonstrated the contextual determinants of ER strategy effectiveness. For example, the controllability of stressors^9^, socioeconomic status^28^ or current goals^25,29^ were shown to influence ER success. Other studies demonstrated situational influences on the choice of ER strategies^26^: when the negativity of stimuli was low, cognitive demand was low, and when long-term goals were activated, participants preferred to choose reappraisal, whereas when the negativity of stimuli was high, cognitive demand was high, and when short-term goals were activated, participants preferred to choose distraction. However, in these studies, ER effectiveness was not measured and ER strategies were not manipulated. In a more recent study, the interaction of strategy and stimuli negativity predicted prefrontal brain region reactivity^30^.

The notion of incorporating individual differences into research on ER strategy effectiveness has also gained some attention. For example, the use and effectiveness of specific strategies were linked to attachment styles^31^, emotional intelligence^32^, dispositional sensitivity to emotional cues (especially prevalent in affective disorders)^19,33^, action orientation^34^ or cognitive control abilities^35,36^.

There is a growing consensus that flexible ER is crucial for the identification, prevention and treatment of affective disturbances that are present in many affective disorders^2,5,19,21^. Greater ER flexibility seems to be associated with better quality of life^24^ and functioning among individuals suffering from mental disorders^37,38^.

Despite initial evidence for the benefits of ER flexibility discussed above, we still lack knowledge about the circumstances under which a specific strategy is most effective. We lack studies that systematically include both contextual and individual factors, that test more (than two) ER strategies in the same study, and incorporate non-declarative (apart from self-report) measures (e.g., behavioral, physiological) to investigate complex interactions between these factors, which jointly predict ER effectiveness.

### Present study

#### General aim

We designed a laboratory experiment that attempts to fill this gap in the ER research. We aimed to investigate how features of the emotional situation (low *vs.* high negativity), the strategy used (reinterpretation as a form of reappraisal, distraction, suppression, no regulation-control condition), and individual differences (heart rate variability) as well as their interaction influence ER effectiveness.

#### Heart rate variability

In our study, we investigated heart rate variability (HRV)—an interindividual psychophysiological factor that can contribute to ER effectiveness. It is argued that emotion regulation dysfunctions might be explained by autonomic nervous system (ANS) dysregulation whose functioning can be indexed by HRV. The Neurovisceral Integration Model^39^ assumes that lower variability between heartbeats is associated with emotion regulation difficulties and this baseline variability was proposed as an objective marker of interindividual emotional capacity^40,41^. Specifically, lower resting HRV was initially linked to lower emotional flexibility suggesting that individuals with lower HRV do not adequately modulate their emotional responses in relation to situational demands^42^. Further, a link between low baseline HRV and self-reported general emotion regulation difficulties^43,44^ as well as a positive relationship between baseline HRV and emotion regulation effectiveness^40,45–49^ have been observed. Higher HRV also predicts better cognitive flexibility—better attentional avoidance and cognitive switching—which may contribute to more effective ER^50,51^.

#### Electromyography

To avoid relying solely on self-report data to assess ER effectiveness, we incorporated electromyographical (EMG) measurements into the experiment. EMG measurements are widely used to investigate the behavioral level of emotional responsiveness—they provide us with information about the level of activity of particular facial muscles that are engaged in the emotional expression of certain emotions^52,53^. Specifically, *corrugator supercilii* facial muscle activity measured with EMG is sensitive to the effects of the regulation of emotion via reinterpretation and suppression^54,55^. We chose EMG measurement as this method has shown more consistent results across studies and proved to be a better indicator of ER effectiveness than more context-dependent autonomic responses^56^. Additionally, we measured self-reported success and effortfulness when employing each ER strategy/experimental instruction during the study.

We predicted that: (H1) we would obtain different patterns of the effectiveness of applied ER strategies for dealing with low and high negativity stimuli—ER will be more effective for low negativity; (H2) the effectiveness of using ER strategies will be influenced by the interaction between situational factors (the strength of negativity) and dispositional factors (heart rate variability); (H3) reinterpretation and distraction will be effective for the evaluation of the subjective experience (declared emotional picture appraisal), whereas suppression will be more effective for downregulating emotional expression (EMG response); (H4) higher HRV will be linked to higher ER effectiveness (both on a subjective level and expressive behavior level); (H5) All ER strategies will be appraised as requiring significantly more effort than no regulation in the control condition.

To the best of our knowledge, our study is the first experimental attempt to combine the investigation of the individual (HRV) and contextual factors (and interaction of both) to show their influence on the effectiveness of several different ER strategies (reinterpretation, distraction, suppression).

## Method

### Participants

A total of 69 adult female participants (aged between 18 and 61 years old: M = 31.12; SD = 11.45) took part in the study. During the time we had to conduct the experiment we managed to find that many volunteers who registered to participate. This number is not very different from other studies in the field. In a recent meta-analysis of the physiological consequences of using emotion regulation strategies, the analyzed studies included between 9 and 168 participants (n analyzed), with most of the studies having fewer than 50 participants^56^.

After removing participants with missing behavioral or psychophysiological data, 54 participants were included in the final analysis. They were recruited via the PESA system at Humboldt University. Following some other researchers^57^, we chose to include only women so as to avoid gender-related factors that might influence emotional responding^58,59^ including HRV^60^ and emotion regulation^61,62^. Based on their preferences, participants received either course credits or financial remuneration (10 euros) for participation in the study.

### Procedure

After participants arrived at the lab, they were welcomed and given the written description of the study (“Information for participants”) as well as the participation agreement—all participants were asked to, first, read them carefully and then sign the consent form if they agreed to participate. Next, participants filled out self-reported measures that are not a part of the current analysis. In the next step, participants were prepared for the experiment, including EKG and EMG recordings.

Before proceeding with the experiment, participants were once again reminded that data acquired from them would stay anonymous and that they could withdraw at any time during the experiment. In case of withdrawal, participants were informed that they would still receive full payment for participation in the experiment. After the preparation stage was completed, the experimental procedure was run using the E-prime software (Psychology Software Tools, 2016)—the procedure can be divided into three phases, following similar procedures employed during previous studies on ER effectiveness^63–65^. These three phases were: (1) Baseline, (2) Training, and (3) Experiment.

The baseline phase was employed to level participants’ emotional experience as well as measure baseline HRV, which was used in our analysis. During this phase, participants watched 30 neutral pictures, each exposed for 10 s (the pictures were the same for each participant). Training phase: during the training phase participants received written instructions for 3 ER strategies: reinterpretation, suppression, and distraction. Following the presentation of each instruction, participants were shown three training pictures and they were asked to discuss in detail with the person conducting the experiment how they employed each of the instructions (e.g., they talked aloud about what reinterpretations of the negative stimuli they came up with when applying reinterpretation). When participants had additional questions, needed clarification, or encountered problems, an experimenter gave further instructions, including possible examples of strategy application. This training procedure was based on the available literature and has been applied in many prior studies^26^. Experimental phase: after completing the training, participants began the actual experimental phase. During this phase, participants watched 8 blocks of negative pictures (4 blocks of high negativity pictures and 4 blocks of low negativity pictures). Before each block they were instructed to use a specific emotion regulation strategy: reinterpretation, suppression, distraction, and no regulation control condition). Each of the strategies appeared twice (once for high- and once for low-negativity pictures). In each block, participants viewed a set of 10 negative pictures and 2 neutral pictures (neutral pictures were used to avoid the expectancy effect) in a randomized order. After each of the blocks, participants answered questions about the subjective appraisal of the valence of the pictures (used in our analyses as an indicator of ER effectiveness: perceived picture negativity): *How negative were the pictures in the last series?* Answer scale between 0 (*Not negative at all*) to 7 (*Extremely negative*). Participants were also asked additional questions about the subjective level of implementing each experimental instruction (used in analyses as subjective effectiveness): “*How successful was the instructed strategy ([Name of the strategy]) in reducing your negative feelings*?”, with an answer scale between *0* (*Not at all*) to *7* (*Very much*). Lastly, we also asked participants about the subjective effortfulness of applying each experimental instruction (used in analyses as subjective effortfulness): *How effortful was following the instructions?* Answer scale between 0 (*Not effortful at all*) to 7 (*Extremely effortful*). The questions were based on previous research on ER effectiveness^64^.

**Figure 1:**
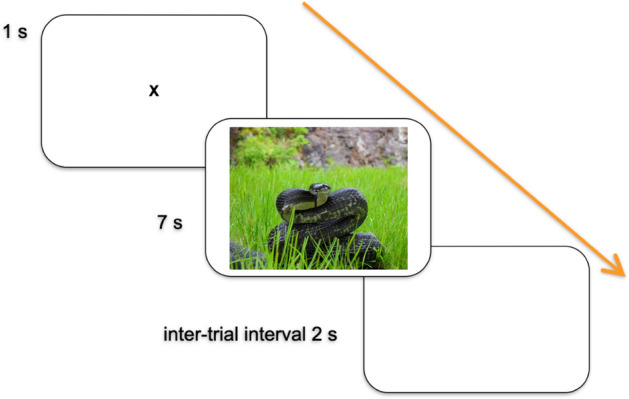
Example of a single experimental trial. The picture originates from the Open Affective Standardized Image Set^92^ and serves only as an example.

A single experimental trial consisted of a fixation point (appearing for 1 s), and 7-s picture exposure (similar to previous studies employing 6 to 8-s exposures, see for example: Bernat et. al., 2011 or McRae et al., 2014), which was then followed by a blank screen for 2 s. Pictures were displayed across the entire screen of a computer monitor. Figure 1 below shows a scheme of an experimental trial.

### Experimental stimuli

Pictures from the International Affective Picture System (IAPS)^66^ were used to elicit affect. The set of pictures was used based on numerous previous studies on ER effectiveness^26,57,65,67–69^. IAPS pictures for high versus low negativity conditions were used based on their valence rating obtained during the validation of the set^66^. We chose pictures similar in arousal ratings but differing in valance. The chosen pictures were grouped into 8 sets, 4 sets for high negativity and 4 sets for low negativity (10 negative and 2 neutral pictures in each set). We made sure the sets were balanced for the content of the photos, for example, 1 war scene in each set, one picture of a child, etc. During the experimental phase, for every participant the sets were randomly paired with experimental instructions/strategies and presented to participants. The sequence of pictures in each set was random for each participant as well. IAPS numbers of all pictures used in the current experiment are listed in the supplementary materials (Tables S1–S3).

### Experimental instructions

When giving participants instructions to follow in each experimental condition, we used instructions similar to those adopted in a wide range of previous research^61,64^. When using reinterpretation, participants were instructed to interpret the situations presented in the pictures so that they had a neutral meaning and caused as few emotions as possible. When using distraction, participants had to focus their attention on or think about something other than the content of the picture. For suppression, participants were instructed to focus on not showing what they felt or thought during the pictures’ presentation. They were instructed to adopt a neutral facial expression and make as few expressions as possible. In the no regulation control condition participants were instructed to just watch the content of the picture without attempting to control their emotions^27,61,64^. The full text of instructions for each experimental condition is available in the supplementary materials.

Lastly, after the experimental phase had been completed, 10 positive IAPS photos were presented (for 7 s each), to reduce the negative emotional state of participants possibly induced by the experimental photos and repair potential negative mood. At this stage the procedure ended, participants were thanked for taking part in the study, debriefed, and rewarded for participation.

Additionally, several questionnaire measures which are not part of the current analysis were also gathered during the study. For transparency reasons, more information on these measures, along with descriptive and correlation statistics, are presented in the supplementary material. The completed questionnaire measures included: Psychological Wellbeing (PWB) Scale^70–72^, The Difficulties in Emotion Regulation Scale (DERS)^73,74^, and Big-Five Factor Markers from the International Personality Item Pool (IPIP)^75^. During the study, electrodermal activity data were also gathered. Results of the analysis of this type of data are not a part of the current work—the analysis was part of a Bachelor’s thesis available online in full and in English here: [blinded link]. No other work was published using the dataset.

#### Ethics

The study procedures were carried out in accordance with the Declaration of Helsinki. The study protocol (including measures and procedure) was approved by the Ethics Committee of the Faculty of Psychology, University of Warsaw. All participants were properly informed about the purpose and procedure of the study and signed an informed consent form before participation.

#### Signal acquisition and data pre-processing

EMG was measured with 4 mm EasyCap GmbH Ag/AgCl miniature surface electrodes filled with Signa gel (Parker Laboratories, Inc.) and a MindWare 8-Slot BioNex bioamplifier (MindWare Technologies, Ltd.). The skin was cleansed with lemon prep peeling and 70% alcohol, impedances were kept below 30 kΩ wherever possible. Raw EMG data were sampled r at 1000 Hz and a gain of 1000, with a 50 Hz notch filter on. The signals were band-pass filtered between 30 and 300 Hz, rectified, and smoothed. Artifacts (e.g., coughing, sneezing) were cleaned manually. Within-subject z-transformed difference scores (trial—baseline) were calculated for each trial to control for individuals' general expressiveness (their general level of facial activity). For the analyses, the signal from each muscle was averaged over each experimental block.

ECG was measured with foam electrodes (insert brand) and the amplifier mentioned above. The skin was cleansed with 70% alcohol. Electrodes were placed as a lead-II configuration, in which a ground electrode was placed at the left clavicle, a negative lead was placed at the right clavicle, and a positive lead was placed below the left rib cage. Recall that due to the ECG recording, a six-minute baseline video was presented, with the last 5 min used for baseline HRV. Raw ECG data were sampled at and a gain of 500, with a 50 Hz notch filter on. The signals were filtered with a low cutoff at 0.5 and a high cutoff at 45 (baseline/muscle noise filter). VLF band was 0.0030, 0.0400, LF band was 0.0400, 0.1200, and HF/RSA band was 0.1200, 0.4000. Window function was Hamming. Respiratory Sinus Arrhythmia (RSA) was calculated based on spectral analysis. Moreover, the root-mean square of successive differences (RMSSD) was calculated by the automatic peak detection algorithm in MindWare BioLab 3.0^76^. Thus, scores were gathered with the help of algorithmic approaches in BioLab whenever possible and invalid data points due to artifacts (e.g., heavy movement) were integrated manually using the BioLab graphic interface denoted for this case. Respiration was tracked via a respiration belt to assist in this process.

### General analysis plan

All analytical procedures were performed in the R statistical environment^77^. To investigate our hypotheses, a Bayesian linear mixed-effects models were estimated (with subject-level random intercepts) to determine if *corrugator supercilii* activity (Model 1), perceived picture negativity (Model 2), subjective effectiveness (Model 3) and subjective effortfulness (Model 4) in applying experimental instructions differed depending on the ER condition, stimulus negativity, and baseline HRV level. The design for each of the models was as follows: 2 (affect: low or high; within-subjects) × 4 (emotional regulation condition: reinterpretation, suppression, distraction, and control; within-subjects) × 2 (HRV group: low or high; between-subjects). The models were fitted using Markov Chain Monte Carlo simulation, as implemented in the *rstanarm* package^78^, with weakly informative priors (see Goodrich et al. [2020] and accompanying code for details). Four chains with 40,000 iterations (20,000 warm-up; thinning rate of 1) were used to estimate each model. The convergence of each model was assessed by inspecting the *R̂* statistic and effective sample size (ESS)^79,80^ associated with each parameter, which were between 1 and 1.05 (*R̂*) and greater than 2000 (ESS) in all cases, indicating no convergence issues. Further downstream inference was performed using the *emmeans* package^81^. To obtain high and low HRV groups we have used a median split strategy (see more in Limitations and Future Directions section).

The analysis plan consisted of two phases. In phase 1, we investigated the differences in experienced stimuli negativity as evidenced by declarative and *corrugator* responses. To this end, separate models with the abovementioned specification were fitted to declarative and psychophysiological data. In phase 2 we investigated the differences in perceived effectiveness and effortfulness of the different emotion regulation strategies. Similar to phase 1, in phase 2 separate models were fitted to explain the effectiveness and effortfulness of appraisals. Estimated means as well as detailed results of statistical comparisons are provided below, as well as in the supplementary information (Tables S1–S3).

Prior to estimating the models, all numeric variables, i.e., effectiveness, effortfulness, and negativity rating responses, as well as the *corrugator supercilii* response, were rescaled to a range between 0 and 1. We used 95% Highest Density Credible Intervals (95% CI) to characterize estimate uncertainty and considered small to be the minimal interesting effect size. Accordingly, a contrast is considered significant if its respective 95% CI does not include zero (and nonsignificant if zero is included). In all post hoc comparisons, a multiplicity adjustment was applied using the multivariate *t* distribution with the same covariance structure as the estimates to determine the adjustment, as implemented in the *emmeans* package^81^. To maintain readability, detailed reports of the obtained results, i.e., model diagnostics, estimates, and contrasts for all pairwise comparisons for *corrugator* activity, as well as behavioral responses, are available in the supplementary materials or accompanying code.

## Results

Firstly, we present descriptive statistics (Table 1) along with correlation indices (Table 2) for all continuous variables included in the current analysis. Of note, higher *corrugator supercilii* activity was related to higher appraisal of picture negativity and lower subjective effectiveness of following experimental instructions. Higher appraisal of picture negativity was also correlated with lower subjective effectiveness, as well as higher effortfulness in following experimental instructions. Moreover, subjective effectiveness had an inverse relation with the perceived effortfulness of following experimental instructions (see Table 2).

**Table 1:** Summary statistics of the collected measures.

Measure	Statistic	
	Median (MAD)	Mean (SD)
Corrugator	− 0.09 (0.96)	− 0.03 (0.92)
Negativity	5.00 (1.48)	4.77 (1.86)
Effectiveness	6.00 (1.48)	5.72 (1.66)
Effortfulness	3.00 (1.48)	3.26 (1.94)
HRV	40.83 (21.75)	48.52 (36.04)

Median (MAD), median value with corresponding median absolute deviation. Mean (SD), mean value with corresponding standard deviation.

**Table 2:** Matrix of Pearson’s correlations between collected measures.

Measure	HRV	Corrugator	Negativity	Effectiveness
Corrugator	0.01			
Negativity	0.05	0.20***		
Effectiveness	0.12*	− 0.10*	− 0.35***	
Effortfulness	0.01	0.07	0.34***	− 0.61***

***, *p* < 0.001; **, *p* < 0.01; *, *p* < 0.05.

### *Corrugator supercilii* activity

**Figure 2:**
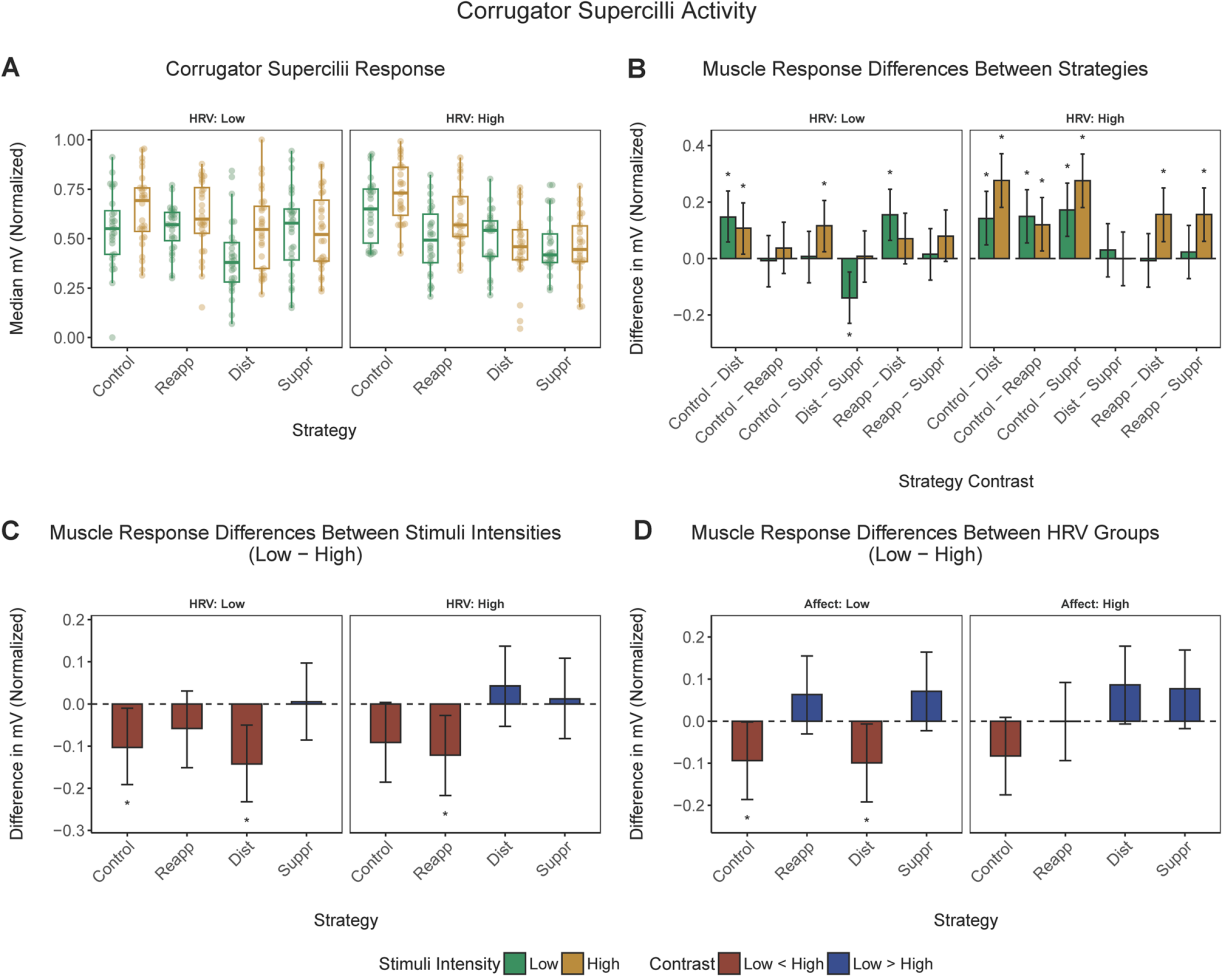
*Corrugator supercilii* activity. (**a**) *Corrugator supercilii* response (median) corresponding to low or high negativity stimuli in different emotion regulation conditions and low or high baseline HRV groups. (**b**) Pairwise contrasts of the *corrugator supercilii* response between emotional regulation conditions corresponding to low or high negativity stimuli in the low or high HRV group. (**c**) Contrasts of the *corrugator supercilii* response between low and high negativity stimuli across emotional regulation conditions in the low or high HRV group. (**d**) Contrasts of the *corrugator supercilii* response between low versus high baseline HRV groups. *Corrugator supercilii* activity is expressed in millivolts (mV) and has been rescaled to a range between 0 and 1. Error bars represent 95% CI of the corresponding estimate. *, significant contrast.

Illustrated in Fig. 2 is the *corrugator supercilii* activity (Panel A), recorded in response to low or high negativity stimuli for each emotion regulation condition, along with the corresponding contrasts between experimental conditions (Panel B), high versus weak emotional stimuli (Panel C) and high versus low baseline HRV groups (Panel D) depending on other factors.

#### Comparing emotion regulation conditions

As seen in Fig. 2 (panel B), low HRV participants showed lower *corrugator supercilii* responses to weak and strong negative stimuli in the distraction condition, compared to the control, as well as lower responses in suppression than in the control condition following exposure to highly negative stimulation. *Corrugator supercilii* response was also lower in distraction than suppression or reinterpretation for low negativity stimuli.

In high HRV participants, the corrugator response was lower in the distraction, reinterpretation, and suppression strategy, compared to the control condition, for both low and high negativity stimuli. Also, in response to high negativity stimuli, the response was lower for distraction and suppression rather than reinterpretation.

#### Low versus high negativity stimuli

Pairwise comparisons between low versus high negativity stimuli showed that low HRV participants responded more to highly negative stimuli in the control and distraction condition, whereas high HRV participants responded more in the reinterpretation condition (Fig. 2, Panel C).

#### Low versus high baseline HRV

Lastly, pairwise comparisons for low versus high baseline HRV groups showed that after viewing low negativity images, high HRV participants showed more *corrugator* activity in the distraction condition or when not regulating their responses (i.e., in the control condition) than the low HRV participants (Fig. 2, Panel D).

### Perceived picture negativity

**Figure 3:**
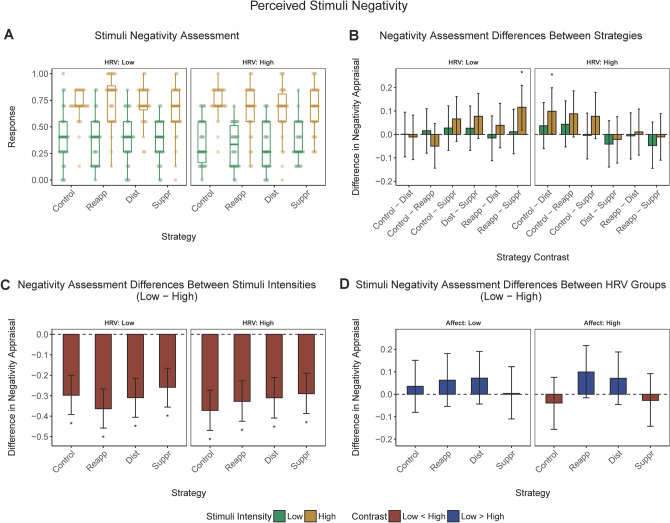
*Perceived picture negativity*. (**a**) *Perceived picture negativity* (median) corresponding to low or high negativity stimuli in different emotion regulation conditions and low or high baseline HRV groups. (**b**) Pairwise contrasts of the *perceived picture negativity* between emotional regulation conditions corresponding to low or high negativity stimuli in the low or high HRV group. (**c**) Contrasts of the *perceived picture negativity* between low and high negativity stimuli across emotional regulation conditions in the low or high HRV group. (**d**) Contrasts of the *perceived picture negativity scores* between low versus high baseline HRV groups. Behavioral responses have been rescaled to a range between 0 and 1. Error bars represent 95% CI of the corresponding estimate. *, significant contrast.

In the next step, we conducted a corresponding analysis for our second main dependent variable—subjective evaluation of stimuli negativity. Figure 3 illustrates median results for perceived picture negativity in response to low or high negativity stimuli (panels A) along with the corresponding pairwise comparisons: between emotion regulation conditions (Panel B), high versus low negativity stimuli (Panel C) and high versus low baseline HRV participant groups (Panel D).

#### Comparing emotion regulation conditions

As seen in Fig. 3 (panel B), low HRV participants rated the high negativity stimuli as more negative in the reinterpretation than in the suppression condition. By contrast, high HRV participants perceived the high negativity stimuli as less negative in the distraction condition compared to the no regulation control condition. No other between-condition comparisons were significant.

#### Low versus high negativity stimuli

High negativity stimuli were perceived as more negative regardless of the HRV group or emotional regulation condition (Fig. 3, panel C).

#### Low versus high baseline HRV

For both low and high negativity stimuli, there were no differences in reported negativity ratings between low and high baseline HRV participants (Fig. 3, panel D).

### Additional analyses 1: subjective effectiveness of ER strategies

**Figure 4:**
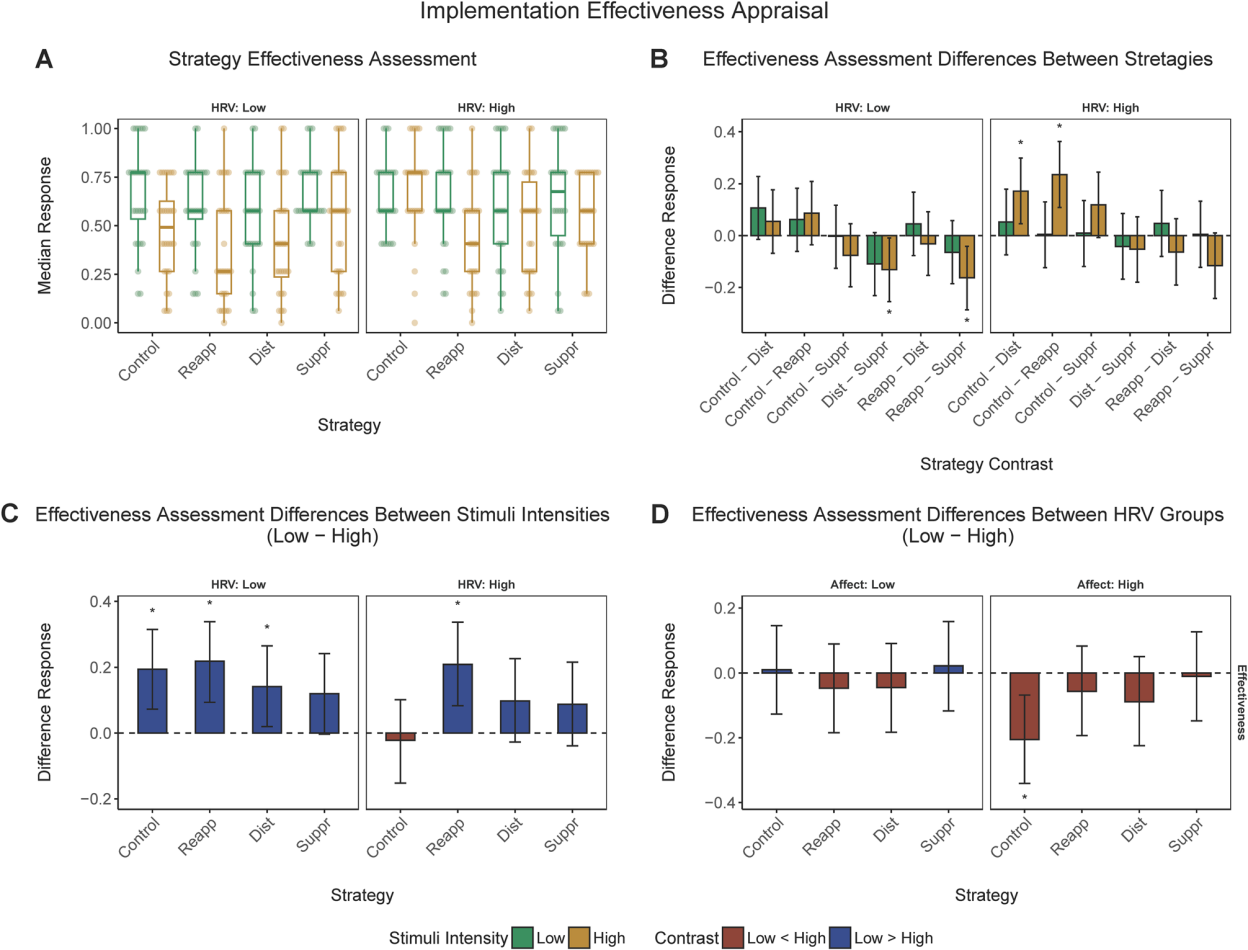
*Perceived effectiveness in applying experimental instructions*. (**a**) *Perceived effectiveness in applying experimental instructions* (median) corresponding to low or high negativity stimuli in different experimental conditions and low or high baseline HRV groups. (**b**) Pairwise contrasts of the *Perceived effectiveness in applying experimental instructions* between emotional regulation conditions corresponding to low or high negativity stimuli in the low or high HRV group. (**c**) Contrasts of the *Perceived effectiveness in applying experimental instructions* between low and high negativity stimuli across emotional regulation conditions in the low or high HRV group. (**d**) Contrasts of the *Perceived effectiveness in applying experimental instructions* between low versus high baseline HRV groups. Behavioral responses have been rescaled to a range between 0 and 1. Error bars represent 95% CI of the corresponding estimate. *, significant contrast.

Participants’ assessments of their effectiveness in using the ER strategies as instructed are illustrated in Fig. 4.

#### Comparing emotion regulation conditions

Perceived effectiveness of the ER strategies varied with both baseline HRV and stimulus negativity (Fig. 4, panel B). Specifically, for high negativity stimuli, low HRV participants perceived themselves as more effective when using the suppression than distraction or reinterpretation strategy. Also for high intensity stimuli, high HRV participants considered themselves to be less effective in following instructions using the distraction or reinterpretation strategy compared to the control condition (no regulation).

#### Low versus high negativity stimuli

As shown in panel C of Fig. 4, low HRV participants considered themselves more effective in following ER instructions in response to low compared to high negativity stimuli when using the reinterpretation or distraction strategies, as well as in the control condition. For high HRV participants, this difference was significant only for the reinterpretation strategy.

#### Low versus high baseline HRV

High HRV participants perceived their effectiveness in following ER instructions in the control condition in response to high negativity stimuli as higher than low HRV participants. No other pairwise comparisons reached significance (Fig. 4, Panel D).

### Additional analyses 2: perceived effort associated with employing emotion regulation strategies

**Figure 5:**
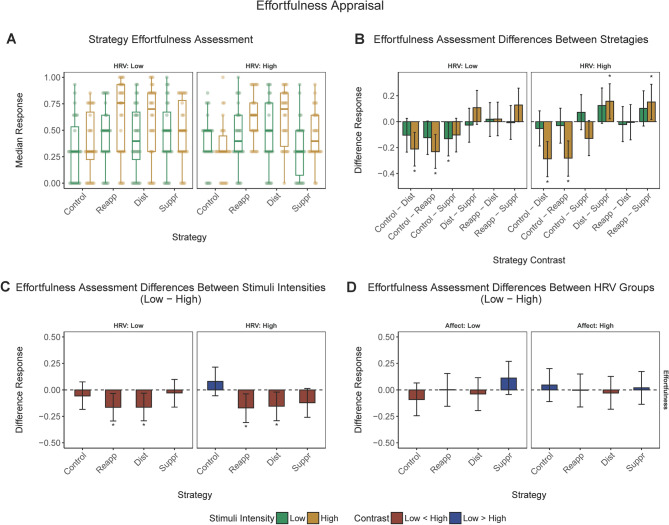
*Perceived effortfulness*. (**a**) *Perceived effortfulness scores* (median) corresponding to low or high negativity stimuli in different experimental conditions and low or high baseline HRV groups. (**b**) Pairwise contrasts of the *Perceived effortfulness* between emotional regulation conditions corresponding to low or high negativity stimuli in the low or high HRV group. (**c**) Contrasts of the *Perceived effortfulness* between low and high negativity stimuli across emotional regulation conditions in the low or high HRV group. (**d**) Contrasts of the *Perceived effortfulness* between low versus high baseline HRV groups. Behavioral responses have been rescaled to a range between 0 and 1. Error bars represent 95% CI of the corresponding estimate. *, significant contrast.

Participants’ assessment of effort involved in emotion regulation depending on experimental condition, negativity, as well as low versus high baseline HRV, are depicted in Fig. 5.

#### Comparing emotion regulation conditions

Pairwise comparisons of the effortfulness across emotional regulation conditions indicated that low HRV participants found the reinterpretation and distraction strategy to be more effortful than the control for high negativity stimuli, as well as the suppression strategy for low negativity stimuli only (Fig. 5, Panel B).

High HRV participants found the distraction and reinterpretation strategy in response to high negativity stimuli to be more effortful than the control task (no regulation). They have also found the suppression strategy to be more effortful than distraction and reinterpretation for highly negative stimuli (Fig. 5, panel B).

#### Low versus high negativity stimuli

Both low and high HRV participants assessed distraction and reinterpretation as more effortful following stimuli higher in negativity (vs. low negativity stimuli; Fig. 5, panel C).

#### Low versus high baseline HRV

There were no significant pairwise differences in the effortfulness of emotion regulation strategies between high versus low baseline HRV groups (Fig. 5, Panel D).

## Discussion

The main aim of the current study was to investigate if and how ER effectiveness on the level of (1) subjective report and (2) emotional expression is affected by the characteristics of a situation in which ER takes place (low *vs.* high emotional negativity), individual differences (low *vs.* high baseline HRV level) and the emotion regulation strategy (reinterpretation, suppression, distraction, and no regulation control condition). Subjective effectiveness and perceived effort of applying the experimental instructions were also investigated. The results suggest that there are, indeed, substantial differences in ER effectiveness depending on these factors. As such, the data provides partial support for the flexible ER framework. However, a subset of the results (e.g., mixed effects of comparisons between emotion regulation strategies for perceived picture negativity) are also somewhat contrary to our predictions, and merit further discussion in the context of the flexible emotion regulation literature.

### Comparisons between emotion regulation conditions

Comparisons between emotion regulation strategies revealed that for high HRV participants, distraction, suppression and reinterpretation all significantly decreased corrugator activity compared to the control condition for both high and low stimulus negativity (partly confirming H4). That is, these participants showed an effective regulation of their emotional expression. Additionally, distraction and suppression were only more effective than reappraisal at downregulating emotional expression for highly negative photos. This suggests that although all the strategies were effective, distraction and suppression worked better to reduce expression. For low HRV participants distraction was most effective in decreasing corrugator responses in the low negativity condition, whereas distraction and suppression decreased emotional expression more than control condition for high negativity stimuli. This pattern of results suggests, firstly, that only high HRV participants were able to effectively use reinterpretation for downregulating emotional expressions. This is consistent with the notion that baseline HRV reflects general emotion regulation capacity^40,41^, as reinterpretation (as a form of reappraisal) is the most complex of the analyzed strategies and likely required the highest regulation abilities and resources^17,27,82,83^. In most cases, distraction and suppression led to a better reduction in emotional expression (while in H3 we hypothesized that only suppression would). Distraction offers a simple means to disengage from unpleasant stimuli whereas the stimulus has to be actively processed when using reinterpretation. Suppression, in turn, was the only strategy that directly targeted emotional expression and not an emotional experience, so it is not surprising that it significantly affected expression. This is also consistent with the literature indicating that suppression is especially effective in regulating behavioral aspects of emotion and can be more effective than reinterpretation at achieving this goal^17,27^.

Yet, even though all strategies could be shown to be effective to some degree, the pattern described above shows that the relative effectiveness depends on both baseline HRV and stimulus negativity (which confirms H2).

Thus, reinterpretation as a more complex strategy was most effective when used by participants who have a higher capacity to regulate their emotional responses (high baseline HRV)^63,84^. By contrast, low HRV participants were less able to use reinterpretation as effectively, suggesting that reinterpretation does not seem to be uniformly effective. This is in line with research pointing to contextual predictors of reappraisal (not stating which specific tactics or forms of reappraisal) success^85^, but contrary to claims suggesting general reappraisal benefits^17,86^ (H3 partly confirmed only for high HRV participants).

Further, when instructed to simply watch the emotional material and show their emotions naturally, high HRV participants were more emotionally expressive than low HRV participants (in the low negativity condition, similar difference was noted for high negativity condition, although it didn’t reach significance), but when instructed to influence their internal states with ER strategies, high HRV participants did this overall more effectively than low HRV ones (again, confirmation of H4). This suggests that in some circumstances high HRV participants could regulate their emotions more flexibly and had less trouble engaging and disengaging from emotion regulation. This is in line with previous research that showed the connection between HRV level and emotion regulation flexibility^42,50,51^. However, as noted, the differences between high and low HRV groups were not evident for suppression as this is a relatively simple ER strategy (as compared to different forms of reappraisal) and thus required fewer resources to implement effectively^27,40,41,87^. This notion is also at least partially supported by the self-reports on the effectiveness and effortfulness of the strategies reported below.

The effect of emotion regulation on the perceived negativity of the stimuli also varied with stimulus negativity and used emotion regulation strategy yet to a much lower degree. Across all experimental conditions, the difference in perceived negativity between high and low negativity stimuli remained significant. However, due to the way in which the question was asked, participants may have reported more on their appraisal of the nature of the stimulus (a cognitive task) and less on how negative the stimulus made them feel. Only the latter aspect should be amenable to emotion regulation.

Both stimulus negativity and baseline HRV affected the perceived effectiveness and effortfulness of emotion regulation in the expected ways. Regulating responses to highly intense stimuli (vs low intensity stimuli) was perceived to be more effortful in the reinterpretation and distraction conditions (partly confirming H5). It was also perceived as less effective, but less so for individuals with high baseline HRV.

Notably, for low and high HRV participants, reinterpretation was deemed especially hard to implement successfully when negativity was high. This is consistent with research indicating that when offered a choice of ER strategy for high negativity material, participants prefer other strategies over reappraisal, which is not the case for weakly stimulating material^88^.

Interestingly, in the high negativity condition, high HRV participants perceived their effectiveness in applying the experimental instruction for the no regulation control condition as higher than low HRV participants. Previously, we discussed that under those circumstances, high HRV participants in fact showed higher emotional expression than low HRV participants. This is consistent with the notion that the ability to express one’s emotion when one is instructed to simply watch the negatively stimulating material, can also be a signal of flexible emotion regulation, as not only effective engagement but also disengagement from self-regulation processes is a signal of regulatory flexibility^19,42,51,89^. Participants who in general should be more effective in regulating their emotion (higher in baseline HRV), had to put effort into not regulating when instructed to just watch the emotional material.

### Implications for theory and practice

The present results have implications both for research and practice. Emotion regulation training should accommodate the fact that the effectiveness of ER strategies is not always the same. Instead, tools used to regulate emotion should be adopted on a case by case basis, accounting for changing circumstances and ER goals, and should also be dependent on the intraindividual characteristics of a person who controls their emotions. For future studies, researchers should focus more on dispositional and contextual factors in investigating the effectiveness of ER strategies^15,18^. Specifically, it may be the case (both for the training of ER skills, as well as research) that low HRV participants require more training for complex ER strategies.

### Limitations and future directions

The current study had some limitations. As noted above, we did not directly ask participants about the level of felt emotions, but rather asked for the perceived valence of the stimuli. The results suggest that this question does not capture potential changes in the feeling state. Moreover, only women participated in this study—future studies should expand our findings to men. Moreover, we used a median split to create low and high HRV groups, and while we are aware that this approach has its disadvantages, it has also been suggested that the use of dichotomization in factorial designs, such as ours, is acceptable^90^.

Additionally, whenever possible, future studies should also incorporate more ecological ways of evoking and studying ER flexibility (for example, using ecological momentary assessment data)—this would allow information to be gathered on ER that occurs in daily life, not only in a laboratory setting.

To obtain a fuller picture of ER flexibility, subsequent studies should expand the current findings by (1) also including additional ER strategies such as acceptance^91^, as well as (2) using a wider range of dependent variables (including neurophysiological and neuroimaging methods, which were not a part of the current study) and (3) expanding the analysis to positive emotion.

## Supplementary Information

Supplementary Information 1.

Supplementary Information 2.

## Data Availability

Dataset and accompanying code for the current paper is available in the Open Science Framework repository: https://osf.io/v8ngw/?view_only=94de4f9cc4504040bdd76acfb2285535.
