# Supplementary figures and images for: Effectiveness of emotion regulation strategies measured by self-report and EMG as a result of strategy used, negative emotion strength and participants’ baseline HRV
Source: Sci Rep. 2023 Apr 17;13:6226. doi: 10.1038/s41598-023-33032-2 (PMC10110539; doi:10.1038/s41598-023-33032-2)

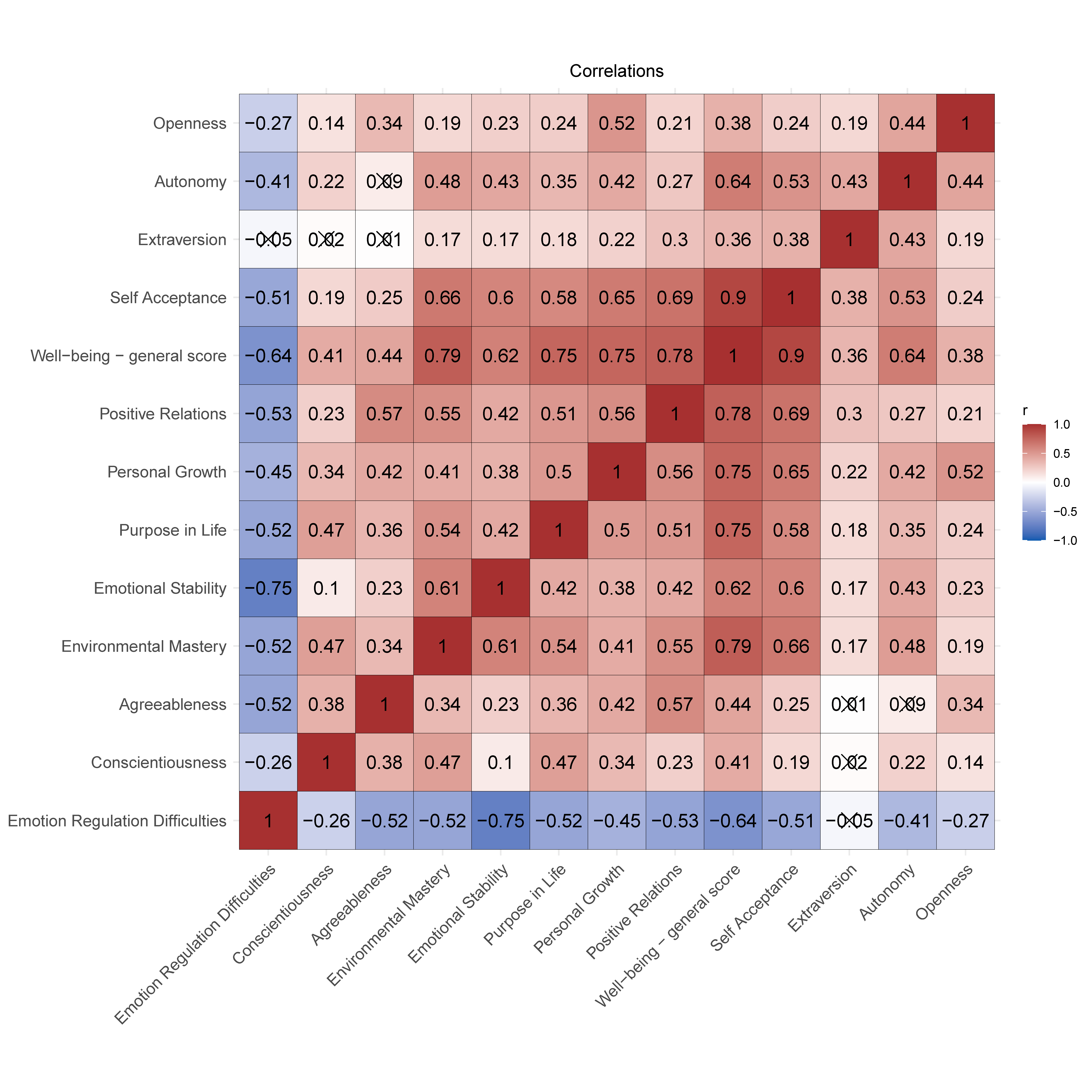

Supplement: Supplementary file 1 — Supplementary Information 1. [file 41598_2023_33032_MOESM1_ESM.png]
