# Supplementary material for: Effectiveness of emotion regulation strategies measured by self-report and EMG as a result of strategy used, negative emotion strength and participants’ baseline HRV
Source: Sci Rep. 2023 Apr 17;13:6226. doi: 10.1038/s41598-023-33032-2 (PMC10110539; doi:10.1038/s41598-023-33032-2)
Supplement: Supplementary file 2 — Supplementary Information 2. [file 41598_2023_33032_MOESM2_ESM.docx]

# Supplementary Materials

## Stimuli used

Index numbers of IAPS photos that were used during the study (numbers refer to the IAPS database):

Training Pictures: 1310, 6571, 7380, 9041, 9042, 9180, 9190, 9220, 9520 (Mean valance: 2.79, SD: 1.38; Mean arousal: 5.34, SD: 2.12)

Neutral Baseline Pictures: 2579, 2580, 52001, 5593, 5740, 5800, 5891, 5900, 7009, 7035, 7090, 7095, 7100, 7130, 7140, 7150, 7175, 7185, 7186, 7187, 7205, 7217, 7235, 7491, 7493, 7496, 7500, 7545, 7550, 7950 (Mean valance: 5.41, SD: 1.35; Mean arousal: 3.10, SD: 2.04)

Experimental phase: highly negative pictures:

*Block 1*: 2053, 2981, 3000, 3030, 3230, 3530, 6230, 6313, 6821, 9181, 9300, 9421 (Mean valance: 1.52, SD: 1.37; Mean arousal: 6.54, SD: 2.12)
 plus neutral pictures: 2190, 7006 (Mean valance: 5.0, SD: 1.06; Mean arousal: 2.54, SD: 1.8)

*Block 2*: 2141, 2800, 3051, 3180, 3181, 3301, 6315, 6415, 6831, 9250, 9250, 9561, 9800 (Mean valance: 1.89, SD: 1.29; Mean arousal: 5.96, SD: 2.26)
 plus neutral pictures: 2280, 7020 (Mean valance: 4.46, SD: 1.3; Mean arousal: 3.07, SD: 1.87)

*Block 3*: 3010, 3059, 3213, 3350, 6212, 6350, 6510, 9040, 9252, 9432, 9571 (Mean valance: 1.59, SD: 1.11; Mean arousal: 6.12, SD: 1.98)
 plus neutral pictures: 2320, 7040 (Mean valance: 5.74, SD: 1.18; Mean arousal: 3.05, SD: 2.01)

*Block 4*: 2900, 3015, 3150, 3261, 6243, 6550, 6560, 8230, 9060, 9253, 9635.1, 9910 (Mean valance: 1.8, SD: 1.26; Mean arousal: 6.32, SD: 2.32)
plus neutral pictures: 2383, 7080 (Mean valance: 4.95, SD: 1.16; Mean arousal: 3.02, SD: 1.89)

Experimental phase: weakly negative pictures:

*Block 1*: 1050, 1300, 2095, 2205, 2590, 2681, 2700, 6190, 6940, 9102, 9280, 9415 (Mean valance: 2.86, SD: 1.63; Mean arousal: 5.32, SD: 2.14)
 plus neutral pictures: 2393, 7000 (Mean valance: 4.99, SD: 1.08; Mean arousal: 4.49, SD: 2.02)

*Block 2*: 1051, 1301, 2120, 2312, 2683, 2710, 6570, 7359, 9006, 9120, 9340, 9440 (Mean valance: 2.7, SD: 16; Mean arousal: 5.64, SD: 2.12)
 plus neutral pictures 2480, 7004 (Mean valance: 4.95, SD: 1.25; Mean arousal: 5.88, SD: 2.16)

*Block 3*: 1111, 1274, 1930, 2130, 2375.1, 2750, 3160, 3300, 6010, 6830, 9160, 9470 (Mean valance: 2.8, SD: 1.39; Mean arousal: 5.48, SD: 2.02)
 plus neutral pictures: 2560, 7010 (Mean valance: 5.78, SD: 0.98; Mean arousal: 2.77, SD: 2.05)

*Block 4*: 1201, 1321, 2490, 2691, 2810, 3220, 6211, 6836, 7360, 9000, 9290, 9622 (Mean valance: 2.92, SD: 1.61; Mean arousal: 5.98, SD: 1.98)

plus neutral pictures: 2575, 7025 (Mean valance: 5.06, SD: 1.12; Mean arousal: 3.61, SD: 1.93)

## Experimental instructions presented to participants for the different emotional regulation strategies

**Reinterpretation:** “*What emotions we experience is to a large extent influenced by the way we interpret situations we are in at the moment. For example, the same situations, in a different time of our life, may seem serious or trivial, annoying or neutral, which influences emotions we subsequently feel. During this part of an experiment you will be watching pictures that may induce different emotional reactions. While watching them carefully try to interpret the situations presented in the pictures so that they have as neutral meaning and caused as little emotions as it is possible. For example, you can think about positive consequences of events presented in the pictures – that everything ended well, despite the fact that the situation looks as negative. Use interpretations that make emotions you experience weaker. Remember that it is very individual if a given interpretation of a situation is effective in reducing your emotions and use interpretations which appeal to you, regardless of how how they can work for others. Follow these instructions for all the pictures presented in this part of a study.*”

**Distraction:** “*One of the way of managing our emotions is trying to concentrate your attention on something else then an emotional situation. During this part of an experiment you will be watching pictures that may induce different emotional reactions. Watch them carefully but at the same time try to think about something else that is not related to the content of the pictures. Shift your attention away from what is making you emotional. For example you may imagine something or recall something that is less emotional then the pictures you watch. Follow these instructions for all the pictures presented in this part of a study.*”

**Suppression:** “*Emotions have many different aspects, for example what you feel, how you behave or how your body reacts. Sometimes when controlling our emotions we focus on certain aspects, for example we don’t what to show what we feel or think. During this part of an experiment you will be watching pictures that may induce different emotional reactions. While watching them carefully do not show your emotions. Try to adopt a neutral facial expression and don’t make any expressions, so that someone observing you couldn’t guess what you feel. Your face and gestures shouldn’t show any signs of emotions you are experiencing. Try to behave in such a way that a person watching you couldn’t guess what you feel. Follow these instructions for all the pictures presented in this part of a study.*”

**Just watch (no regulation control condition):** “*During this part of the experiment, you will be watching pictures that may induce different emotional reactions. Please take a close look at the pictures without using any specific way to influence your emotions, just look at the pictures without following any specific strategy and let your emotions happen. Follow these instructions for all the pictures presented in this part of a study.*”

## Estimate details

**Table S1.** Estimates and contrasts across HRV groups, emotional regulation conditions, and stimuli negativity levels for *corrugator supercilli* activity, stimuli negativity appraisal, as well as appraisal of strategy effectiveness and effortfulness. Reported are estimated median values with their 95% Confidence Intervals.

| **Response** | **HRV** | **Strategy** | **Low** | **High** | **Contrast** |
| --- | --- | --- | --- | --- | --- |
| Corrugator | Low | Control | 0.55 [0.48, 0.61] | 0.65 [0.59, 0.72] | -0.10 [-0.19, -0.01] * |
|  |  | Reappraisal | 0.55 [0.49, 0.62] | 0.61 [0.55, 0.68] | -0.06 [-0.15, 0.03] |
|  |  | Distraction | 0.40 [0.34, 0.47] | 0.54 [0.48, 0.61] | -0.14 [-0.23, -0.05] * |
|  |  | Suppression | 0.54 [0.47, 0.60] | 0.53 [0.47, 0.60] | 0.01 [-0.09, 0.10] |
|  | High | Control | 0.64 [0.57, 0.71] | 0.73 [0.67, 0.80] | -0.09 [-0.19, 0.00] |
|  |  | Reappraisal | 0.49 [0.42, 0.56] | 0.61 [0.55, 0.68] | -0.12 [-0.22, -0.03] * |
|  |  | Distraction | 0.50 [0.43, 0.57] | 0.46 [0.39, 0.52] | 0.04 [-0.05, 0.14] |
|  |  | Suppression | 0.47 [0.40, 0.54] | 0.46 [0.39, 0.53] | 0.01 [-0.08, 0.11] |
| Negativity | Low | Control | 0.40 [0.32, 0.48] | 0.69 [0.61, 0.77] | -0.30 [-0.39, -0.20] * |
|  |  | Reappraisal | 0.38 [0.30, 0.46] | 0.74 [0.67, 0.83] | -0.36 [-0.46, -0.27] * |
|  |  | Distraction | 0.40 [0.31, 0.48] | 0.70 [0.62, 0.79] | -0.31 [-0.41, -0.21] * |
|  |  | Suppression | 0.37 [0.29, 0.45] | 0.63 [0.55, 0.71] | -0.26 [-0.36, -0.17] * |
|  | High | Control | 0.36 [0.28, 0.44] | 0.73 [0.65, 0.82] | -0.37 [-0.47, -0.27] * |
|  |  | Reappraisal | 0.32 [0.23, 0.40] | 0.64 [0.56, 0.73] | -0.33 [-0.42, -0.23] * |
|  |  | Distraction | 0.32 [0.24, 0.41] | 0.63 [0.55, 0.72] | -0.31 [-0.41, -0.21] * |
|  |  | Suppression | 0.36 [0.28, 0.45] | 0.65 [0.57, 0.74] | -0.29 [-0.39, -0.19] * |
| Effectiveness | Low | Control | 0.66 [0.57, 0.76] | 0.47 [0.38, 0.56] | 0.19 [ 0.07, 0.31] * |
|  |  | Reappraisal | 0.60 [0.51, 0.70] | 0.38 [0.29, 0.48] | 0.22 [ 0.09, 0.34] * |
|  |  | Distraction | 0.56 [0.46, 0.65] | 0.42 [0.32, 0.51] | 0.14 [ 0.02, 0.26] * |
|  |  | Suppression | 0.67 [0.57, 0.76] | 0.55 [0.45, 0.64] | 0.12 [ 0.00, 0.24] |
|  | High | Control | 0.65 [0.55, 0.75] | 0.68 [0.58, 0.78] | -0.02 [-0.15, 0.10] |
|  |  | Reappraisal | 0.65 [0.55, 0.75] | 0.44 [0.34, 0.54] | 0.21 [ 0.08, 0.34] * |
|  |  | Distraction | 0.60 [0.50, 0.70] | 0.51 [0.41, 0.60] | 0.10 [-0.03, 0.23] |
|  |  | Suppression | 0.65 [0.55, 0.75] | 0.56 [0.46, 0.65] | 0.09 [-0.04, 0.22] |
| Effortfulness | Low | Control | 0.32 [0.21, 0.43] | 0.37 [0.27, 0.48] | -0.06 [-0.18, 0.08] |
|  |  | Reappraisal | 0.44 [0.33, 0.55] | 0.61 [0.50, 0.71] | -0.16 [-0.29, -0.03] * |
|  |  | Distraction | 0.42 [0.31, 0.53] | 0.59 [0.48, 0.69] | -0.16 [-0.29, -0.03] * |
|  |  | Suppression | 0.45 [0.34, 0.56] | 0.48 [0.37, 0.59] | -0.03 [-0.16, 0.10] |
|  | High | Control | 0.41 [0.30, 0.52] | 0.33 [0.22, 0.44] | 0.08 [-0.06, 0.21] |
|  |  | Reappraisal | 0.44 [0.33, 0.55] | 0.61 [0.50, 0.72] | -0.17 [-0.31, -0.04] * |
|  |  | Distraction | 0.46 [0.35, 0.57] | 0.61 [0.50, 0.73] | -0.15 [-0.29, -0.02] * |
|  |  | Suppression | 0.34 [0.22, 0.45] | 0.46 [0.34, 0.57] | -0.12 [-0.26, 0.01] |

*Note.* *, significant contrast as its respective 95% CI does not include zero.

**Table S2.** Pairwise contrasts across emotional regulation conditions for *corrugator supercilii* activity in each HRV group and stimuli negativity condition.

| **Response** | **HRV** | **Affect** | **Contrast** | **Estimate** |
| --- | --- | --- | --- | --- |
| Corrugator | Low | Low | Control - Reappraisal | -0.01 [-0.10, 0.08] |
|  |  |  | Control - Distraction | 0.15 [ 0.06, 0.24] * |
|  |  |  | Control - Suppression | 0.01 [-0.09, 0.10] |
|  |  |  | Reappraisal - Distraction | 0.15 [ 0.06, 0.25] * |
|  |  |  | Reappraisal - Suppression | 0.02 [-0.08, 0.11] |
|  |  |  | Distraction - Suppression | -0.14 [-0.23, -0.05] * |
|  | High | Low | Control - Reappraisal | 0.15 [ 0.06, 0.24] * |
|  |  |  | Control - Distraction | 0.14 [ 0.05, 0.24] * |
|  |  |  | Control - Suppression | 0.17 [ 0.08, 0.27] * |
|  |  |  | Reappraisal - Distraction | -0.01 [-0.10, 0.09] |
|  |  |  | Reappraisal - Suppression | 0.02 [-0.07, 0.12] |
|  |  |  | Distraction - Suppression | 0.03 [-0.07, 0.12] |
|  | Low | High | Control - Reappraisal | 0.04 [-0.05, 0.13] |
|  |  |  | Control - Distraction | 0.11 [ 0.02, 0.20] * |
|  |  |  | Control - Suppression | 0.12 [ 0.02, 0.21] * |
|  |  |  | Reappraisal - Distraction | 0.07 [-0.02, 0.16] |
|  |  |  | Reappraisal - Suppression | 0.08 [-0.01, 0.17] |
|  |  |  | Distraction - Suppression | 0.01 [-0.08, 0.10] |
|  | High | High | Control - Reappraisal | 0.12 [ 0.03, 0.22] * |
|  |  |  | Control - Distraction | 0.28 [ 0.18, 0.37] * |
|  |  |  | Control - Suppression | 0.28 [ 0.18, 0.37] * |
|  |  |  | Reappraisal - Distraction | 0.16 [ 0.06, 0.25] * |
|  |  |  | Reappraisal - Suppression | 0.16 [ 0.06, 0.25] * |
|  |  |  | Distraction - Suppression | 0.00 [-0.10, 0.09] |
| Negativity | Low | Low | Control - Reappraisal | 0.02 [-0.08, 0.11] |
|  |  |  | Control - Distraction | 0.00 [-0.10, 0.09] |
|  |  |  | Control - Suppression | 0.03 [-0.07, 0.12] |
|  |  |  | Reappraisal - Distraction | -0.02 [-0.11, 0.08] |
|  |  |  | Reappraisal - Suppression | 0.01 [-0.08, 0.11] |
|  |  |  | Distraction - Suppression | 0.03 [-0.07, 0.12] |
|  | High | Low | Control - Reappraisal | 0.04 [-0.05, 0.14] |
|  |  |  | Control - Distraction | 0.04 [-0.06, 0.14] |
|  |  |  | Control - Suppression | 0.00 [-0.10, 0.09] |
|  |  |  | Reappraisal - Distraction | -0.01 [-0.11, 0.09] |
|  |  |  | Reappraisal - Suppression | -0.05 [-0.14, 0.05] |
|  |  |  | Distraction - Suppression | -0.04 [-0.14, 0.06] |
|  | Low | High | Control - Reappraisal | -0.05 [-0.14, 0.05] |
|  |  |  | Control - Distraction | -0.01 [-0.11, 0.08] |
|  |  |  | Control - Suppression | 0.07 [-0.03, 0.16] |
|  |  |  | Reappraisal - Distraction | 0.04 [-0.06, 0.13] |
|  |  |  | Reappraisal - Suppression | 0.12 [ 0.02, 0.21] * |
|  |  |  | Distraction - Suppression | 0.08 [-0.02, 0.17] |
|  | High | High | Control - Reappraisal | 0.09 [-0.01, 0.19] |
|  |  |  | Control - Distraction | 0.10 [ 0.00, 0.20] * |
|  |  |  | Control - Suppression | 0.08 [-0.02, 0.18] |
|  |  |  | Reappraisal - Distraction | 0.01 [-0.09, 0.11] |
|  |  |  | Reappraisal - Suppression | -0.01 [-0.11, 0.09] |
|  |  |  | Distraction - Suppression | -0.02 [-0.12, 0.08] |

*Note.* *, significant contrast as its respective 95% CI does not include zero.

**Table S3.** Pairwise contrasts across emotional regulation conditions for effectiveness and effortfulness appraisal in each HRV group and stimuli negativity condition.

| **Response Type** | **HRV Group** | **Affect** | **Contrast** | **Estimate** |
| --- | --- | --- | --- | --- |
| Effectiveness | Low | Low | Control - Reappraisal | 0.06 [-0.06, 0.18] |
|  |  |  | Control - Distraction | 0.11 [-0.01, 0.23] |
|  |  |  | Control - Suppression | 0.00 [-0.13, 0.12] |
|  |  |  | Reappraisal - Distraction | 0.05 [-0.08, 0.17] |
|  |  |  | Reappraisal - Suppression | -0.06 [-0.19, 0.06] |
|  |  |  | Distraction - Suppression | -0.11 [-0.23, 0.01] |
|  | High | Low | Control - Reappraisal | 0.00 [-0.12, 0.13] |
|  |  |  | Control - Distraction | 0.05 [-0.07, 0.18] |
|  |  |  | Control - Suppression | 0.01 [-0.12, 0.14] |
|  |  |  | Reappraisal - Distraction | 0.05 [-0.08, 0.17] |
|  |  |  | Reappraisal - Suppression | 0.00 [-0.12, 0.13] |
|  |  |  | Distraction - Suppression | -0.04 [-0.17, 0.09] |
|  | Low | High | Control - Reappraisal | 0.09 [-0.04, 0.21] |
|  |  |  | Control - Distraction | 0.05 [-0.07, 0.18] |
|  |  |  | Control - Suppression | -0.08 [-0.20, 0.05] |
|  |  |  | Reappraisal - Distraction | -0.03 [-0.15, 0.09] |
|  |  |  | Reappraisal - Suppression | -0.16 [-0.29, -0.04] * |
|  |  |  | Distraction - Suppression | -0.13 [-0.25, -0.01] * |
|  | High | High | Control - Reappraisal | 0.23 [ 0.11, 0.36] * |
|  |  |  | Control - Distraction | 0.17 [ 0.05, 0.30] * |
|  |  |  | Control - Suppression | 0.12 [-0.01, 0.24] |
|  |  |  | Reappraisal - Distraction | -0.06 [-0.19, 0.06] |
|  |  |  | Reappraisal - Suppression | -0.12 [-0.24, 0.01] |
|  |  |  | Distraction - Suppression | -0.05 [-0.18, 0.07] |
| Effortfulness | Low | Low | Control - Reappraisal | -0.12 [-0.25, 0.00] |
|  |  |  | Control - Distraction | -0.10 [-0.23, 0.03] |
|  |  |  | Control - Suppression | -0.13 [-0.26, 0.00] * |
|  |  |  | Reappraisal - Distraction | 0.02 [-0.11, 0.15] |
|  |  |  | Reappraisal - Suppression | -0.01 [-0.14, 0.12] |
|  |  |  | Distraction - Suppression | -0.03 [-0.16, 0.10] |
|  | High | Low | Control - Reappraisal | -0.03 [-0.17, 0.10] |
|  |  |  | Control - Distraction | -0.05 [-0.19, 0.08] |
|  |  |  | Control - Suppression | 0.07 [-0.06, 0.21] |
|  |  |  | Reappraisal - Distraction | -0.02 [-0.15, 0.12] |
|  |  |  | Reappraisal - Suppression | 0.10 [-0.03, 0.24] |
|  |  |  | Distraction - Suppression | 0.12 [-0.01, 0.26] |
|  | Low | High | Control - Reappraisal | -0.23 [-0.36, -0.10] * |
|  |  |  | Control - Distraction | -0.21 [-0.34, -0.08] * |
|  |  |  | Control - Suppression | -0.10 [-0.23, 0.03] |
|  |  |  | Reappraisal - Distraction | 0.02 [-0.11, 0.15] |
|  |  |  | Reappraisal - Suppression | 0.13 [ 0.00, 0.26] |
|  |  |  | Distraction - Suppression | 0.11 [-0.02, 0.24] |
|  | High | High | Control - Reappraisal | -0.28 [-0.42, -0.15] * |
|  |  |  | Control - Distraction | -0.29 [-0.42, -0.15] * |
|  |  |  | Control - Suppression | -0.13 [-0.26, 0.01] |
|  |  |  | Reappraisal - Distraction | -0.01 [-0.14, 0.13] |
|  |  |  | Reappraisal - Suppression | 0.15 [ 0.02, 0.29] * |
|  |  |  | Distraction - Suppression | 0.16 [ 0.02, 0.29] * |

*Note.* *, significant contrast as its respective 95% CI does not include zero.

**Table S4.** Table of descriptive statistics for additional measures collected.

| **Measure** | **Statistic** | |
| --- | --- | --- |
|  | **Median (MAD)** | **Mean (SD)** |
| Emotion Regulation Difficulties - general score | 2.16 (0.48) | 2.23 (0.56) |
| Agreeableness (Personality BIG Five) | 6.35 (1.04) | 6.29 (0.88) |
| Conscientiousness (Personality BIG Five) | 5.85 (0.89) | 5.65 (1.11) |
| Emotional Stability (Personality BIG Five) | 4.70 (1.48) | 4.71 (1.11) |
| Extraversion (Personality BIG Five) | 4.55 (1.41) | 4.64 (1.24) |
| Openness (Personality BIG Five) | 5.95 (0.89) | 5.86 (0.82) |
| Autonomy (Psychological Well-Being) | 4.44 (0.66) | 4.34 (0.71) |
| Environmental Mastery (Psychological Well-Being) | 4.78 (0.58) | 4.62 (0.78) |
| Personal Growth (Psychological Well-Being) | 4.88 (0.56) | 4.72 (0.63) |
| Positive Relations (Psychological Well-Being) | 4.67 (0.99) | 4.65 (0.83) |
| Purpose in Life (Psychological Well-Being) | 4.67 (0.99) | 4.56 (0.8) |
| Self-Acceptance (Psychological Well-Being) | 4.75 (0.89) | 4.55 (0.92) |
| Well-being - general score (Psychological Well-Being) | 4.69 (0.44) | 4.57 (0.6) |

*Note 1.* Median (MAD), median value with corresponding median absolute deviation. Mean (SD), mean value with corresponding standard deviation.

*Note 2.* Table includes the scores for the following scales (and their subscales): Psychological Wellbeing (PWB) Scale^1–3^, The Difficulties in Emotion Regulation Scale (DERS)^4,5^, and Big-Five Factor Markers from the International Personality Item Pool (IPIP)^6^.

**Figures**

**Figure S1.** Matrix of Pearson's r correlations for additional collected measures. Crossed-out coefficients correspond to non-significant (p > .05) relationships.


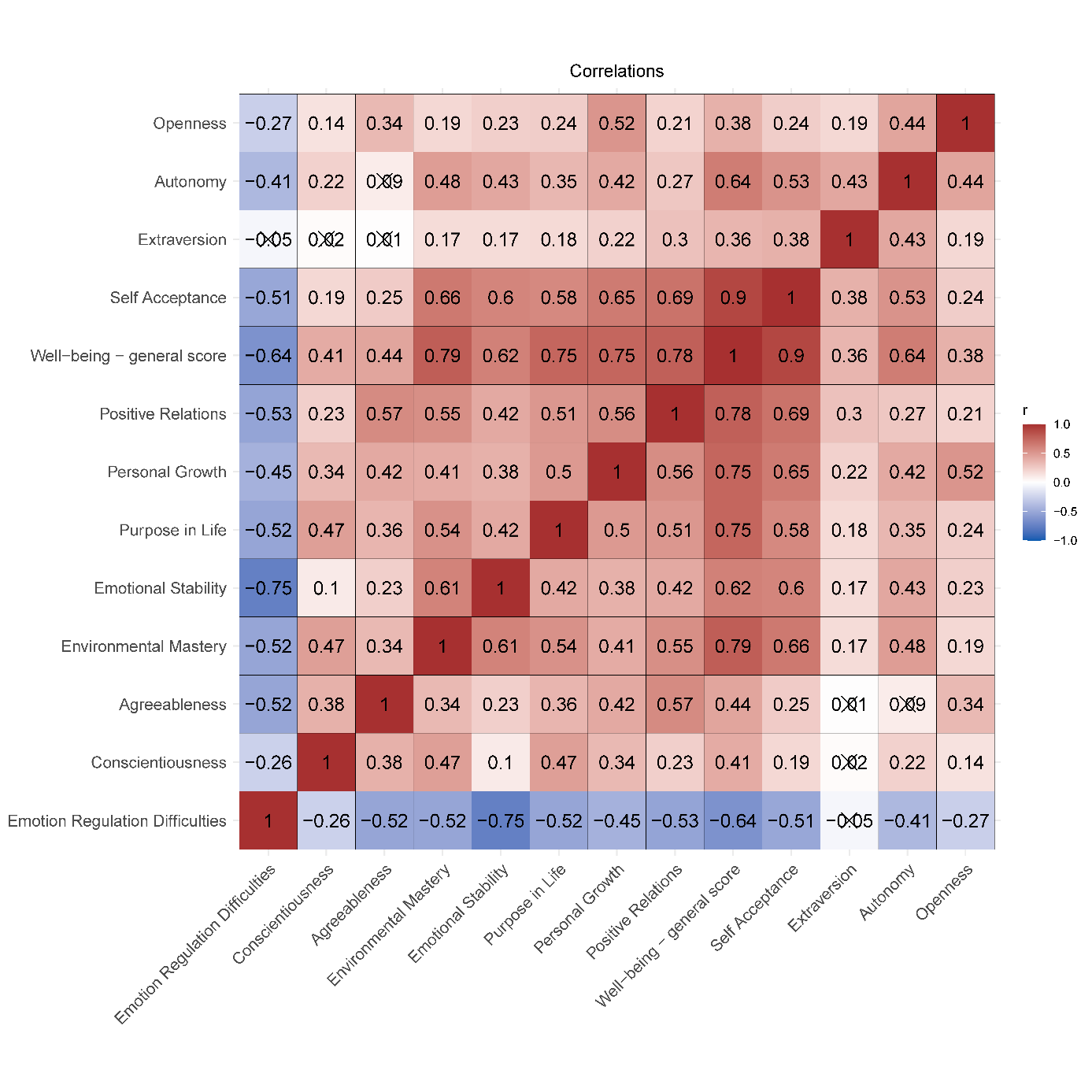


**References**

1. Ryff, C. D. Happiness is everything, or is it? Explorations on the meaning of psychological well-being. *Journal of Personality and Social Psychology* **57**, 1069–1081 (1989).

2. Risch, A., Strohmayer, C. & Stangier, U. Psychologische Wohlbefindensskala-PWS. *Unpublished manuscript* (2005).

3. Ryff, C. D. Psychological Well-Being Revisited: Advances in the Science and Practice of Eudaimonia. *Psychother Psychosom* **83**, 10–28 (2014).

4. Neumann, A., van Lier, P. A. C., Gratz, K. L. & Koot, H. M. Multidimensional Assessment of Emotion Regulation Difficulties in Adolescents Using the Difficulties in Emotion Regulation Scale. *Assessment* **17**, 138–149 (2010).

5. Gratz, K. L. & Roemer, L. Multidimensional Assessment of Emotion Regulation and Dysregulation: Development, Factor Structure, and Initial Validation of the Difficulties in Emotion Regulation Scale. *Journal of Psychopathology and Behavioral Assessment* **26**, 41–54 (2004).

6. Goldberg, L. R. The development of markers for the Big-Five factor structure. *Psychological Assessment* **4**, 26–42 (1992).
